# Fibroblast Growth Factor 19 Increases the Excitability of Pre-Motor Glutamatergic Dorsal Vagal Complex Neurons From Hyperglycemic Mice

**DOI:** 10.3389/fendo.2021.765359

**Published:** 2021-11-11

**Authors:** Jordan B. Wean, Bret N. Smith

**Affiliations:** ^1^ Department of Physiology, College of Medicine, University of Kentucky, Lexington, KY, United States; ^2^ Department of Neuroscience, College of Medicine, University of Kentucky, Lexington, KY, United States

**Keywords:** diabetes, fibroblast growth factor, hyperglycemia, vagus nerve, EPSC, area postrema, nucleus tractus solitarius, parasympathetic

## Abstract

Intracerebroventricular administration of the protein hormone fibroblast growth factor 19 (FGF19) to the hindbrain produces potent antidiabetic effects in hyperglycemic mice that are likely mediated through a vagal parasympathetic mechanism. FGF19 increases the synaptic excitability of parasympathetic motor neurons in the dorsal motor nucleus of the vagus (DMV) from hyperglycemic, but not normoglycemic, mice but the source of this synaptic input is unknown. Neurons in the area postrema (AP) and nucleus tractus solitarius (NTS) express high levels of FGF receptors and exert glutamatergic control over the DMV. This study tested the hypothesis that FGF19 increases glutamate release in the DMV by increasing the activity of glutamatergic AP and NTS neurons in hyperglycemic mice. Glutamate photoactivation experiments confirmed that FGF19 increases synaptic glutamate release from AP and NTS neurons that connect to the DMV in hyperglycemic, but not normoglycemic mice. Contrary to expectations, FGF19 produced a mixed effect on intrinsic membrane properties in the NTS with a trend towards inhibition, suggesting that another mechanism was responsible for the observed effects on glutamate release in the DMV. Consistent with the hypothesis, FGF19 increased action potential-dependent glutamate release in the NTS in hyperglycemic mice only. Finally, glutamate photoactivation experiments confirmed that FGF19 increases the activity of glutamatergic AP neurons that project to the NTS in hyperglycemic mice. Together, these results support the hypothesis that FGF19 increases glutamate release from AP and NTS neurons that project to the DMV in hyperglycemic mice. FGF19 therefore modifies the local vago-vagal reflex circuitry at several points. Additionally, since the AP and NTS communicate with several other metabolic regulatory nuclei in the brain, FGF19 in the hindbrain may alter neuroendocrine and behavioral aspects of metabolism, in addition to changes in parasympathetic output.

## Introduction

Fibroblast growth factor 19 (FGF19) is an ileal-derived protein hormone that produces potent, anti-diabetic, and anti-obesogenic effects. Although early work suggested that these effects were mediated mainly by FGF19 acting on peripheral targets, several reports have now found that acute intracerebroventricular (ICV) administration of FGF19 may act at multiple distinct sites in the brain to regulate energy balance. Lateral ventricle administration of FGF19 increases metabolic rate (1), while 3^rd^ ventricular administration was found to decrease food intake, lower insulin resistance, improve glucose tolerance, and reduce blood glucose concentrations in rodent models of diabetes and obesity (2–5). These findings suggest a hypothalamic mechanism since FGF19 was found to suppress AGRP/NPY activity (4) and decrease plasma ACTH (2). However, we demonstrated that 4^th^ ventricular administration of FGF19 also decreases blood glucose in type 1 diabetic (T1DM) mice, suggesting the hindbrain as an underappreciated target tissue for this system (6).

The brainstem dorsal vagal complex (DVC) is an important homeostatic regulatory center that tightly controls parasympathetic output in response to numerous convergent inputs, both neuronal and humoral. The DVC is principally comprised of the area postrema (AP), the nucleus tractus solitarius (NTS), and the dorsal motor nucleus of the vagus (DMV). The NTS integrates vagal afferent, viscerosensory information with input from several brain areas, including the hypothalamus and AP (7–10). In turn, the NTS regulates neural activity in the DMV through both glutamatergic and GABAergic projections (11, 12). Finally, DMV neurons project cholinergic outputs through the efferent vagus nerve to regulate hepatic glucose production, gastric motility, and pancreatic exocrine secretion, among other visceral regulatory functions (13–18). Additionally, the AP and NTS contain fenestrated capillaries that permit the passage of humoral components that may be excluded by the blood-brain barrier elsewhere (19, 20). DVC neurons respond to many primary metabolic hormones including insulin, leptin, ghrelin, glucagon, GLP-1 (21–25) and importantly, FGF19 (6). The DVC, especially the AP and NTS, contains multiple FGF receptors (FGFR) as well as ß-klotho, an obligate co-receptor (26–28), suggesting that neuronal activity in the DVC may regulate metabolic homeostatic mechanisms in response to endogenous FGF signaling.

In a mouse model of type 1 diabetes (T1DM), FGF19 consistently and robustly increased action potential-dependent excitatory synaptic transmission to the DMV (6). This suggests that FGF19 increases the activity of glutamatergic neurons immediately afferent to the DMV, which remain intact in the slice preparation. A likely source of these glutamatergic inputs is the NTS since the NTS to DMV connection is well documented (12, 29–31) and is open to modulation by various other peptides and neurotransmitters (31–34). Interestingly, receptor expression data suggest that the AP could also be a target for FGF19 since the AP expresses significantly more FGFR than surrounding areas (28) and expresses more ß-klotho and FGFR2 than any other brain area, in addition to high levels of FGFR1 and FGFR3 (26). Moreover, glutamatergic AP neurons project extensively to the NTS and DMV (35). Taken together, these findings suggest that FGF19 may act on intrinsic DVC circuitry to modify synaptic input to the DMV and consequently modulate vagally-mediated glucoregulation.

This study tests the hypothesis that FGF19 increases excitatory neurotransmission in the DVC by altering excitability of NTS and AP neurons. In addition to functioning within local DVC circuits, the NTS and AP communicate bidirectionally with other brain areas thought to be involved in regulating energy metabolism (7, 8, 36). Thus, FGF19-mediated alteration of neuronal activity in these areas is likely to have wide-reaching metabolic effects by modulating vago-vagal circuit dynamics as well as influencing more distal areas that control ingestive behaviors and regulate energy balance through autonomic or neuroendocrine means.

## Materials and Methods

### Animals

All mice used for experiments were juvenile (3-8 weeks old) male and female FVB mice (FVB-Tg(GadGFP)4570Swn/J, FVB; Jackson Laboratory, Bar Harbor, ME, USA). This mouse expresses enhanced green fluorescent under the control of the GAD67 promoter and allows for the visual identification of GABAergic neurons. Animals were housed under 14:10 light-dark conditions in the University of Kentucky Division of Laboratory Animal Resources facilities with food and water available ad-libitum. Approximately equal numbers of both sexes were used and results from both sexes were aggregated. All animal procedures were approved by the University of Kentucky Animal Care and Use Committee.

To destroy insulin-secreting pancreatic β-cells, mice were given an intraperitoneal injection of streptozotocin (STZ; 200 mg/kg in 0.15mL of 0.1 M citric acid; Sigma-Aldrich, St. Louis, MO, USA) after a 6-hour fast. After injection, mice were returned to their home cages and blood glucose was monitored daily by tail lance (Nova Max Plus, Nova Diabetes Care, Billerica, MA, USA). Mice were used for experiments after ≥5 days of continual hyperglycemia (≥ 300 mg/dL). It has been previously established that a similar period of hyperglycemia is sufficient to produce lasting changes in both synaptic and intrinsic properties of NTS and DMV neurons (37–42). Mice that displayed persistent hyperglycemia after STZ injection were considered a model of T1DM.

### Electrophysiology

Mice were anesthetized using isoflurane inhalation (confirmed using foot pinch response) and decapitated at between 1000 and 1200 hrs. The brain was then rapidly removed and placed in ice-cold, oxygenated (2-4°C; 95% O_2_/5% CO_2_) artificial cerebrospinal fluid (ACSF). In all experiments (except where the addition of drugs is noted), ACSF was composed of (in mM): 124 NaCl, 3 KCl, 26 NaHCO_3_, 1.4 NaH_2_PO_4_, 11 glucose, 1.3 CaCl_2_, and 1.3 MgCl_2_. The hindbrain was mounted to a sectioning stage using cyanoacrylate glue and submerged in ACSF. Coronal brainstem slices (300 µm) were made using a vibratome (Series 1000; Technical Products International, St. Louis, MO, USA). After cutting, slices were incubated in a holding chamber for 1 hour in warmed (30°C-35°C), oxygenated ACSF.

For recordings, slices were transferred to a recording chamber on a fixed-stage, upright microscope (BX51WI; Olympus, Melville, NY, USA) and superfused with warmed ACSF (32-34°C). Whole-cell patch-clamp recordings were performed under visual control and cells were identified using infrared illumination with differential interference contract optics. Glass recording pipettes (1.65 mm OD, 1.2 mm ID; King Precision Glass, Claremont, CA, USA) were filled with internal solution that contained (in mM): 130 K-gluconate, 1 NaCl, 5 EGTA, 10 HEPES, 1 MgCl2, 1 CaCl2, 3 KOH, and 2 Mg-ATP; pH = 7.2-7.3, adjusted with 5 M KOH. Recordings were made using a Multiclamp 700B amplifier, Digidata 1440A digitizer, and pClamp 10.6 software (Molecular Devices, Axon Instruments, Sunnyvale, CA, USA). Data were recorded at 20 kHz and filtered at 3 kHz.

Added to the ACSF for specific experiments were the following: Fibroblast growth factor 19 (FGF19; 230 pM; ProspecBio, Ness Ziona, Israel), 4-Methoxy-7-nitroindolinyl-caged-L-glutamate (MNI caged glutamate; 250 µM; Tocris/BioTechne, Minneapolis, MN, USA), tetrodotoxin (TTX; 2μM; Alomone Labs, Jerusalem, Israel), and picrotoxin (100 μM; Alomone Labs). The concentration for FGF19 was chosen because we have previously shown that this concentration alters intrinsic and synaptic properties of DMV neurons (6). Additionally, this concentration has been shown to stimulate approximately half-maximal glucose uptake and phosphorylated extracellular signal-related kinase (pERK) induction in cell culture assays (43). FGF19 was applied for 5 minutes and was applied once per slice to avoid any lasting effects of prior applications.

DMV neurons were identified by morphology (elongated, tear-drop-shaped soma ≥ 20 µm) and by their location in the slice (located along the ventral edge of the DVC). NTS neurons were also identified by morphology and location in slice (dorsal to the DMV). The mouse model used here expresses enhanced green fluorescent protein (EGFP) under a GAD67 promoter. This allows for visual identification of a large proportion of GABAergic NTS neurons. Since previous research suggested that FGF19 altered glutamatergic, but not GABAergic transmission in the DVC, EGFP-negative NTS neurons were targeted to increase the likelihood of recording from a glutamatergic neuron (6).

Once whole-cell configuration was achieved, neurons were held near their resting membrane potential (RMP) for at least 5 minutes to allow proper equilibration of the cytoplasm and pipette solution. Acceptable series resistance was considered to be <25 MΩ (range = 6.044-24.89 MΩ; mean = 13.21 ± 0.36 MΩ) and was regularly monitored; recordings were discarded if series resistance or cell capacitance changed by ≥ 20% during recording. All voltage values were corrected *post-hoc* for the liquid junction potential (calculated at -15 mV). Excitatory postsynaptic currents (EPSCs) were recorded at -85 mV, the approximate reversal potential for Cl^-^, to prevent interference by inhibitory currents. For continuous recordings of EPSCs, 2 min of activity was analyzed. Input resistance (R_in_) was calculated as the slope of the line that best fit the points produced by 500 ms negative current injections ranging from -20 pA to 0 pA in 5 pA increments. Neurons were considered responsive if FGF19 produced a ≥20% change in R_in_. Current *versus* action potential frequency (I-F) analysis was made in the same group of neurons that were used to measure R_in_ by measuring action potentials resulting from positive current injections ranging from 0 pA to 20 pA in 5 pA increments.

Similar to previous reports of glutamate photoactivation in the DVC (12, 33, 34), MNI-caged glutamate (250 µM) was added to recirculating ACSF and uncaged using 30 ms pulses of UV light (UV filter; Chroma Technology, Rockingham, VT). UV light, controlled by an automated shutter system (Uniblitz VMM-D1, Vincent Associates, USA), was directed to the slice through the 40X water immersion objective. Aperture width was set to minimum to stimulate a small patch of neurons (approximately 75 µm diameter stimulation region). When light was positioned directly over the recorded cell, UV pulses produced large, fast inward currents in voltage-clamp mode and significant depolarization in current-clamp mode (typically >200 pA and >20 mV, respectively). To find extant glutamatergic connections, the objective was systematically moved throughout the AP or NTS until the UV pulse produced a detectable synaptic response. Care was taken not to directly photostimulate the recorded neuron, thus ensuring that any observed response was due to glutamate stimulation of neurons that projected to the recorded cell. Recordings consisted of 10 repetitions of: A one second pre-stimulus period, a 30 ms UV pulse, then a two second post-stimulus period. Results were reported as a difference in EPSC frequency during the 500 ms immediately after stimulation *versus* that in the 1-second period before stimulation. Successful glutamate uncaging was defined as a >1 Hz change in frequency. Mean fold change was obtained by averaging the individual fold-change values of each neuron.

### Statistics and Analysis

Recordings were analyzed using pClamp 10.6 (Axon Instruments), Minianalysis 6.0.7 (Synaptosoft, Decatur, GA, USA), SAS 9.4 (SAS Institute, Cary, NC, USA) and Prism 8 (GraphPad Software, San Diego, CA, USA). Within-cell analysis of multi-event recordings (e.g., EPSCs before and after drug application) was performed using the 2-sample Kolmogorov-Smirnov (K-S) test. Grouped analyses were performed using a paired t-test when one before/after pair was present or repeated measures generalized linear mixed model with Tukey multiple comparisons when multiple before/after pairs were present. Significance was set at p<0.05 for all analyses. Unless otherwise indicated, data are presented as mean ± SEM.

## Results

### FGF19 Increases the Excitability Of Glutamatergic AP and NTS Neurons That Project to the DMV in Hyperglycemic Mice

**Figure 1:**
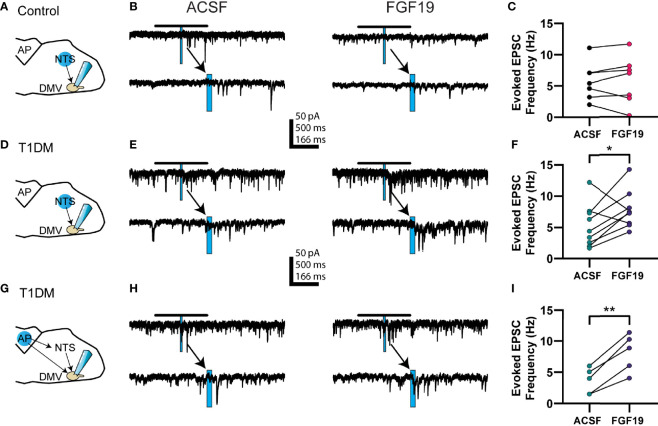
FGF19 increases the excitability of glutamatergic AP and NTS neurons that project to the DMV in hyperglycemic mice. **(A)** Diagram showing a typical stimulation and recording location for cells in **(B, C)** (normoglycemic control mice). **(B)** Representative voltage clamp recordings of evoked EPSCs for the NTS to DMV circuit in control mice. **(C)** Evoked EPSC response in control mice before and after addition of FGF19 (230 pM; n = 7; *p > 0.05). **(D)** Diagram showing a typical stimulation and recording location for cells in **(E, F)** (hyperglycemic mice). **(E)** Representative voltage clamp recordings of evoked EPSCs for the NTS to DMV circuit in hyperglycemic, type 1 diabetic mice (T1DM). **(F)** Evoked EPSC response in this group before and after addition of FGF19 (n = 10; *p < 0.05). **(G)** Diagram showing a typical stimulation and recording location for cells in **(H, I)** (hyperglycemic mice). **(H)** Representative voltage clamp recordings of evoked EPSCs for the AP to DMV circuit in T1DM mice. **(I)** Evoked EPSC response in this group before and after addition of FGF19 (n = 5; **p < 0.01). In representative traces, blue rectangle indicates stimulation time and duration. Arrows point to an expanded trace showing 500 ms before and after stimulation. All cells recorded at -85 mV. In **(A**, **D**, **G)**, the blue circle represents a typical photostimulation region. The actual area of photostimulation was smaller in diameter (~75 µm) than the circle pictured and stimuli were applied to multiple areas in the indicated nucleus. Paired t-test used for all analyses. All recordings performed in the presence of MNI-caged glutamate (250 µM) and picrotoxin (100 µM).

Previous reports found that FGF19 increased action potential-dependent glutamate release in the DMV in hyperglycemic mice (6). Both the NTS and AP remain intact in the slice preparation, express abundant FGF receptors, and project glutamatergic projections to the DMV (26, 28, 35). Thus, it was determined that these areas were the most likely source of excitatory input. To identify the effect of FGF19 on excitatory neurotransmission from the NTS and AP to the DMV, glutamate photostimulation was performed in these areas while recording EPSCs in DMV neurons (**Figure 1**). Briefly, the effect of glutamate uncaging was determined by measuring the difference in EPSC frequency before and after UV light pulses. All recordings were made in the presence of picrotoxin (100 µM), a GABA receptor type-A blocker, to prevent any effects caused by stimulating local GABAergic neurons.

When glutamate photostimulation was performed in the NTS of control mice, FGF19 failed to alter the mean effect of glutamate photostimulation in the NTS on EPSC frequency in the DMV (**Figure 1C**; ACSF: 5.78 ± 1.05 Hz; FGF19: 5.81 ± 1.38 Hz; n = 7; p=0.409). Since there was no overall effect on the response to photostimulation in the NTS, and since there was no effect of FGF19 on sEPSC frequency in DMV neurons from control mice previously (6), no further uncaging experiments were performed in this group. When glutamate photoactivation was performed in the NTS of T1DM mice, FGF19 significantly increased the mean effect of glutamate photostimulation in the NTS on EPSC frequency in the DMV (**Figure 1F**; ACSF: 5.18 ± 1.02 Hz; FGF19: 7.52 ± 0.91 Hz; n = 10; p=0.016). This represented an average 1.84-fold increase in the response. Similarly, when glutamate photolysis was performed in the AP in T1DM mice, FGF19 significantly increased the mean effect of glutamate photostimulation (**Figure 1I**; ACSF: 3.62 ± 0.82 Hz; FGF19: 8.16 ± 1.21 Hz; n = 5; p=0.004). This represented an average 2.6-fold increase in the response. Together, these data suggest that FGF19 increases the excitability of glutamatergic neurons in the AP and NTS that project to the DMV in hyperglycemic mice.

### FGF19 Produces Mixed Effects on Intrinsic Excitability of NTS Neurons

**Figure 2:**
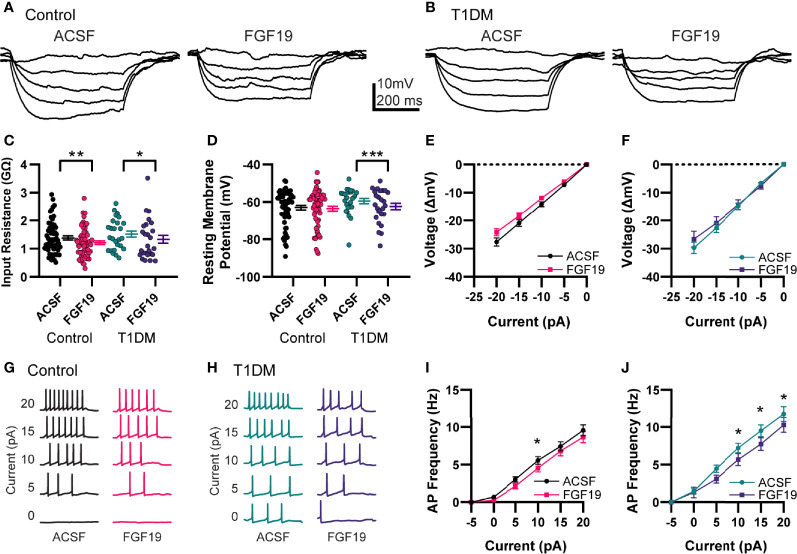
FGF19 produces mixed effects on intrinsic excitability of NTS neurons. Representative current step recordings in current clamp mode from **(A)** control and **(B)** T1DM mice. **(C)** FGF19 significantly decreased mean input resistance in both control (n = 60; **p < 0.01) and T1DM mice (n = 26; *p < 0.05). **(D)** FGF19 did not alter mean resting membrane potential in control mice (n = 60; p > 0.05) but significantly hyperpolarized mean RMP in T1DM mice (n = 26; ***p < 0.001). **(E)** Averaged current-voltage relationship in seen control mice (n = 60). **(F)** Averaged current-voltage relationship seen in T1DM mice (n = 26). Representative traces showing action potential response to positive current injections in control **(G)** and T1DM mice **(H)**. **(I)** Averaged action potential frequency response to positive current injections in control mice (n = 60; p < 0.05). **(J)** Averaged action potential frequency response to positive current injections in T1DM mice (n = 26; p < 0.05). For all panels, * indicates p < 0.05; ** indicates p < 0.01, *** indicates p < 0.001. Repeated measures generalized linear mixed model analysis used for all analyses. No pharmacological blockers were included for these experiments.

To determine the effects of FGF19 on intrinsic excitability of NTS neurons, resting membrane potential (RMP) and input resistance (R_in_) were measured in current-clamp mode (**Figures 2A–F**). Recordings were performed in control and T1DM mice to understand whether hyperglycemia modulates the effect of FGF19 in these neurons as suggested by previous findings (6). A neuron that displayed a >20% change in R_in_ or >2 mV change in RMP was considered responsive to FGF19. In neurons from control mice, FGF19 modestly but significantly decreased mean R_in_ (**Figure 2C**; ACSF: 1.38 ± 0.07 GΩ; FGF19: 1.21 ± 0.07 GΩ; n = 60; p=0.0011). In this group, FGF19 produced a change in R_in_ in approximately 50% of neurons with a trend towards lower Rin (8 increased; 21 decreased; 31 no change). FGF19 did not alter mean RMP in NTS neurons from control mice (**Figure 2D**; ACSF: -63 ± 1.22 mV; FGF19; -63.6 ± 1.29 mV; n = 60; p=0.2279), but individual neurons responded with a change in RMP in similar proportions to those observed with R_in_ (8 increased; 16 decreased; 36 no change). In neurons from T1DM mice, FGF19 produced a small but significant decrease in mean R_in_ (**Figure 2C**; ACSF: 1.51 ± 0.11 GΩ; FGF19: 1.33 ± 0.14 GΩ; n = 26; p=0.0202). In this group, FGF19 altered R_in_ in 50% of neurons with a predominately inhibitory effect (2 increased, 11 decreased, 13 no change). FGF19 also hyperpolarized the mean RMP in this group (**Figure 2D**; ACSF: -59.5 ± 1.37 mV; FGF19: -62.3 ± 1.72 mV; n = 26; p=0.0001). Similarly to R_in_, FGF19 altered RMP in approximately half of neurons (0 increased, 12 decreased, 14 no change). Neither R_in_ nor RMP differed between control and T1DM groups prior to FGF19 application. Moreover, proportions of neurons that responded to FGF19 did not differ between groups for both R_in_ (Control; Responsive: 29; Non-responsive: 31; T1DM; Responsive: 13, Non-responsive: 13; p>0.99; Fisher’s exact test) and RMP (Control; Responsive: 24; Non-responsive: 36; T1DM; Responsive: 12; Non-responsive: 14; p = 0.63; Fisher’s exact test). To identify the effects of FGF19 on action potential responsiveness in the NTS, positive current step recordings were performed in the same neurons to produce evoked action potentials (**Figures 2G**–**J**). In neurons from control mice, FGF19 decreased mean evoked action potential frequency at the 10 pA current step (**Figure 2I**; n = 60; p=0.0253). In neurons from T1DM mice, FGF19 decreased mean evoked action potential frequency at the 10, 15, and 20 pA current steps (**Figure 2J**; n = 26; 10pA, p = 0.0314; 15 pA, p = 0.0135; 20 pA, p = 0.0408). These data suggest that FGF19 produces mixed effects that trend toward inhibition in NTS neurons that responded. Importantly, these effects did not differ between groups.

### FGF19 Increases sEPSC Frequency in NTS Neurons From Hyperglycemic Mice

**Figure 3:**
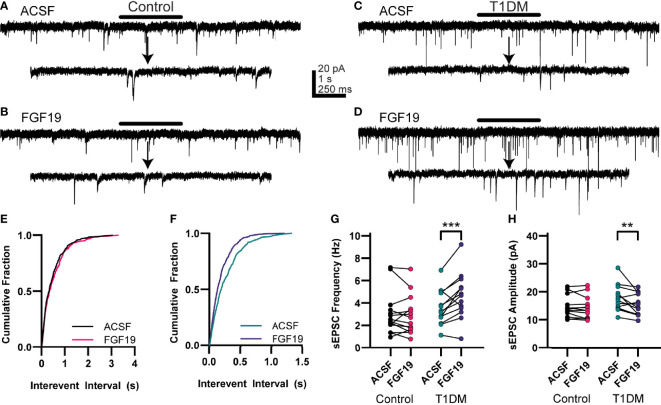
FGF19 increases sEPSC frequency in NTS neurons from hyperglycemic mice. **(A, B)** representative voltage clamp recordings of sEPSCs in NTS neurons from control mice. **(C, D)** Representative voltage clamp recordings of sEPSCs in NTS neurons from T1DM mice. **(E)** Cumulative fraction plot for the traces from **(A, B)**. **(F)** Cumulative fraction plot for the traces from **(C, D)**. **(G)** FGF19 does not alter sEPSC frequency in control mice (n = 14; p > 0.05) but significantly increases sEPSC frequency in T1DM mice (n = 13; ***p < 0.001). **(H)** FGF19 does not alter sEPSC amplitude in control mice (n= 14; p > 0.05) and slightly decreases sEPSC ampltide in T1DM mice (n = 13; **p < 0.01). Arrow indicates 2 s expanded portions indicated by the black bar above the trace. All cells recorded at -85 mV. Repeated measures generalized linear mixed model analysis used for all analyses. No pharmacological blockers were applied for these experiments.

Because FGF19’s effects on intrinsic excitability were inconsistent with its effects on spontaneous EPSC frequency reported previously (6), and since FGF19 consistently increased EPSCs in the DMV after glutamate photolysis in either the NTS or AP, excitatory neurotransmission to NTS neurons was measured. To assess this, NTS neurons were voltage-clamped at -85 mV to record spontaneous EPSCs (sEPSC) before and after bath application of FGF19 (**Figure 3**). In neurons from control mice, FGF19 did not significantly alter mean sEPSC frequency (**Figure 3G**; ACSF: 3.02 ± 0.51 Hz; FGF19: 2.89 ± 0.47 Hz; n = 14; p=0.5940). In this group, FGF19 altered sEPSC frequency in 7 out of 14 neurons (2 increased; 5 decreased; p < 0.02, within recording K-S test). FGF19 also failed to produce effects on mean sEPSC amplitude in this group (**Figure 3H**; ACSF: 14.4 ± 1.04 pA; FGF19: 14.0 ± 1.07 pA; n = 14; p=0.5813). In neurons from T1DM mice however, FGF19 significantly increased mean sEPSC frequency (**Figure 3G**; ACSF: 3.60 ± 0.43 Hz; FGF19: 4.62 ± 0.57 Hz; n = 13; p=0.0004). In this group, FGF19 altered sEPSC frequency in all neurons, with a predominately excitatory effect (11 increased; 2 decreased; p<0.02, K-S test). FGF19 subtly altered mean sEPSC amplitude in this group (**Figure 3H**; ACSF: 17.7 ± 1.31 pA; FGF19: 15.3 ± 1.09 pA; n = 13; p=0.001). Neither sEPSC frequency nor sEPSC amplitude differed between the control and T1DM groups prior to FGF19 application. Together, these data show that FGF19 increases spontaneous excitatory synaptic input to the NTS in T1DM but not normoglycemic mice, similar to our previous findings in the DMV (6).

### FGF19 Does Not Alter mEPSC Frequency in NTS Neurons From Hyperglycemic Mice

**Figure 4:**
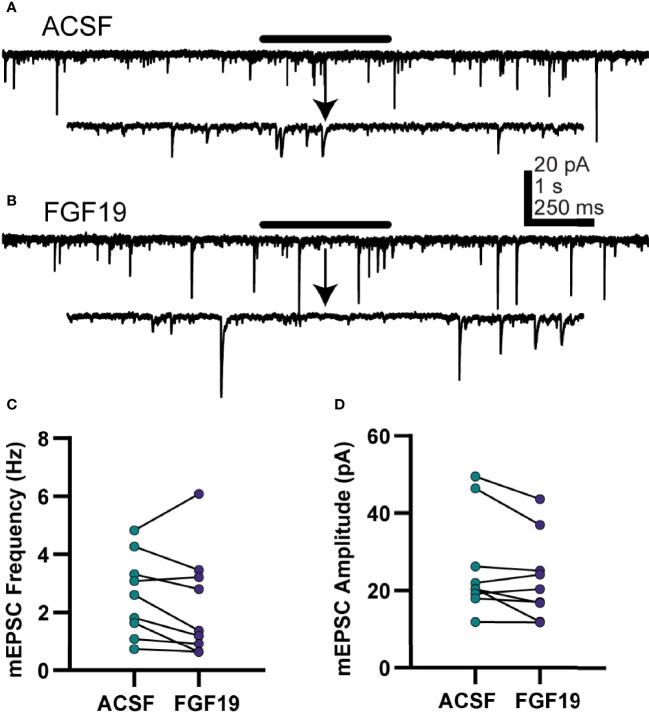
FGF19 does not alter mEPSC frequency in NTS neurons from hyperglycemic mice. **(A, B)** representative voltage clamp recordings in NTS neurons from T1DM mice in the presence of TTX (2 µM). **(C)** FGF19 failed to alter mean mEPSC frequency (n = 9; p > 0.05). **(D)** FGF19 failed to alter mean mEPSC amplitude (n = 9; p > 0.05). All cells recorded at -85 mV. Paired t-test used for all analyses. All recordings performed in TTX (2 µM).

FGF19 increased mean spontaneous excitatory neurotransmission in neurons from T1DM, but not normoglycemic mice, so further experiments were performed to understand the nature of this effect in T1DM mice only. To determine whether the effect on sEPSCs was due to actions at the soma or terminal of the presynaptic neuron, action potential independent, “miniature” excitatory postsynaptic currents (mEPSCs) were recorded (**Figure 4**). NTS neurons from T1DM mice were recorded similarly to the sEPSC recordings above with the addition of TTX (2 µM) to prevent action potentials. Unlike for sEPSCs, FGF19 failed to alter mean mEPSC frequency in DMV neurons from T1DM mice (**Figure 4C**; ACSF: 2.60 ± 0.47 Hz; FGF19: 2.26 ± 0.60 Hz; n = 9; p=0.210). FGF19 also did not alter mean mEPSC amplitude (**Figure 4D**; ACSF: 26.0 ± 4.34 pA; FGF19: 23.1 ± 3.64 pA; n = 9; p=0.071). These data, taken with the sEPSC results above suggest that FGF19 consistently produced a net increase in synaptic excitation of NTS neurons from T1DM mice. Because this increase in sEPSC is abrogated in the presence of TTX, FGF19 likely increases the activity of intact glutamatergic neurons afferent to the NTS.

### FGF19 Increases the Excitability of Glutamatergic AP Neurons That Project to the NTS in Hyperglycemic Mice

**Figure 5:**
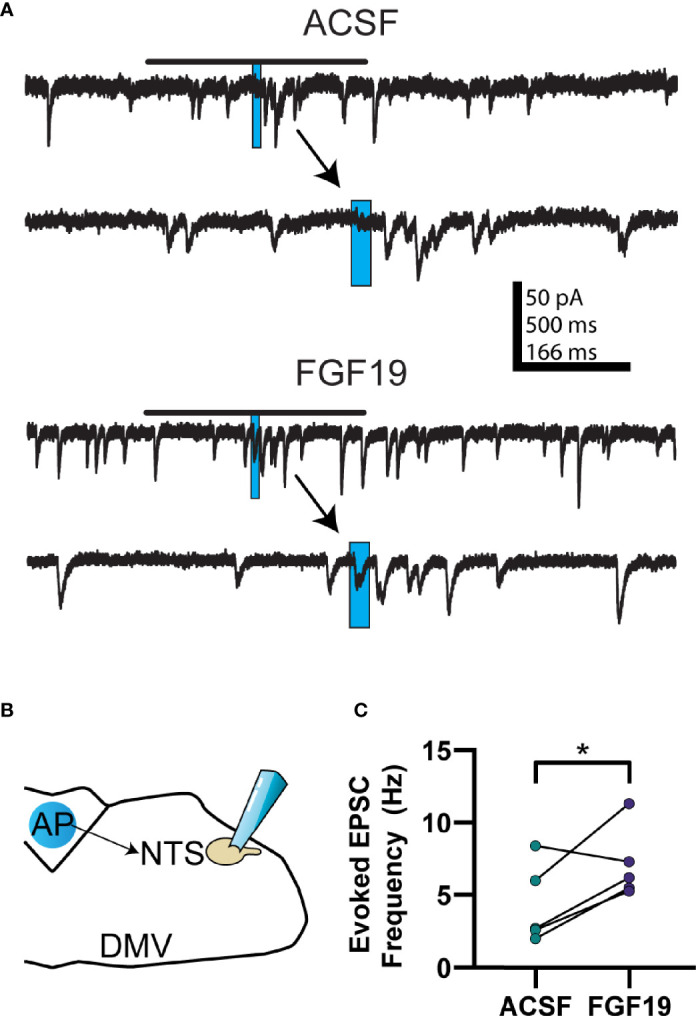
FGF19 increases the excitability of glutamatergic AP neurons that project to the NTS in hyperglycemic mice. **(A)** Representative voltage clamp recordings of evoked EPSCs for the AP to NTS circuit in T1DM mice. **(B)** Diagram showing a typical stimulation and recording regions for cells in **(A, C)**. **(C)** Evoked EPSC response in this group before and after addition of FGF19 (n = 5; *p < 0.05). In representative traces, blue rectangle indicates stimulation time and duration. Arrows point to an expanded trace showing 500 ms before and after stimulation. All cells recorded at -85 mV. Paired t-test used for analysis. All recordings performed in MNI-caged glutamate (250 µM) and picrotoxin (100 µM).

Since FGF19 was found to increase the effect of glutamate photostimulation of AP neurons and since the AP has glutamatergic projections to both the NTS and DMV (35), it was hypothesized that the AP was also a likely source of local excitatory input to the NTS. To identify the effects of FGF19 on glutamatergic neurotransmission from the AP to the NTS, glutamate uncaging was performed to increase activity of AP neurons while recording from neurons in the NTS (**Figure 5**). Because FGF19 was found to increase sEPSC frequency in NTS neurons from T1DM mice only, further glutamate uncaging experiments were restricted to this group of mice. When glutamate photostimulation was performed in the AP, FGF19 significantly increased the mean effect of uncaging on EPSC frequency in the NTS (**Figure 5C**; ACSF: 4.34 ± 1.10 Hz; FGF19: 7.12 ± 0.98 Hz; n = 5; p=0.037). The averaged fold-change of all cells in this group was 1.96. Together, these data suggest that FGF19 increases the excitability of glutamatergic AP neurons that project to the NTS in hyperglycemic mice.

## Discussion

These results indicate that FGF19 increases glutamatergic transmission at multiple points in DVC circuitry in hyperglycemic, but not normoglycemic mice, which implies a mechanistic understanding of FGF19 activity in the hindbrain. We found previously that FGF19 decreased blood glucose in hyperglycemic mice through actions in the dorsal hindbrain, and this effect was abrogated by co-administration of a peripheral muscarinic receptor blocker (6), suggesting a parasympathetic mechanism. Similar to the current findings, we also found that FGF19 altered the excitability of DMV neurons in a complex manner that was heavily influenced by hyperglycemic state. These results suggested that FGF19 functioned in the DVC through a relatively restricted mechanism (i.e., that FGF19 modified the activity of central vago-vagal reflexes, resulting in altered parasympathetic output). The current findings expand upon what was established previously by identifying multiple local, excitatory synaptic circuits within the DVC that are positively modulated by FGF19 to increase glutamate release in the DMV. These results are especially intriguing considering that, while the DMV is typically associated with autonomic regulation, the NTS and AP show extensive interconnectivity with several other brain regions involved in regulating metabolism (8, 35, 36, 44–46). This suggests that FGF19 in the hindbrain may regulate metabolism through multiple mechanisms in addition to autonomic changes, which is consistent with the diverse effects produced by ICV FGF19 reported previously (2–4, 47).

Previous data showed that the effect of FGF19 on EPSC frequency in the DMV was blocked by TTX, suggesting a neuronal source that remained intact within slice preparation. Although the source was unknown, it was hypothesized to be the NTS, since it is a primary source of synaptic input to the DMV and expresses the receptors/co-receptor required to respond to FGF19 (26, 28, 48, 49). However, these same expression data suggest the intriguing hypothesis that the AP may also participate in the response of DMV neurons to FGF19. Indeed, the AP displays significantly higher expression of multiple FGFRs and ß-klotho than the NTS and shows some of the highest expression levels of any brain area (26). Thus, neurons in the NTS and AP were tested regarding the effects of FGF19 on their glutamatergic connections to the DMV.

The glutamate photolysis experiments performed here demonstrated that FGF19 increases the excitatory synaptic input to the DMV arising from both AP and NTS neurons. The involvement of the NTS was predicted, since the excitatory glutamatergic NTS to DMV circuit has been well-characterized (9, 11, 12, 50). However, there is little known about the role of the AP in local vagal circuitry. Interestingly, there is evidence to suggest that the AP may be an unappreciated participant in the canonical vago-vagal reflex circuit. Several early tracing studies (51–54), later confirmed with viral and genetic techniques (35, 55, 56), found that the AP receives vagal sensory input and projects glutamatergic output toward the NTS and DMV. This suggests that the AP may serve as a vagal sensory integration center, in a similar capacity as the NTS. Despite this, electrophysiological studies exploring the role that the AP plays in regulating local DVC circuitry have been few and more work is warranted to understand this connection (57–60).

The current findings confirm that AP neurons can modulate DMV activity *via* glutamatergic synaptic connections and that FGF19 increases the activity of this input. This does not necessarily imply the existence of a monosynaptic connection, since it is possible that the AP regulates DMV activity through intermediary NTS neurons. However, considering the widespread glutamatergic innervation from the AP throughout the DVC and the extensive dendritic fields of DMV neurons, it is likely that the AP communicates to the DMV both mono- and polysynaptically (35, 61). A priori, in light of the AP’s traditional role as the principal emetic center in the brain, the existence of a monosynaptic connection from AP to DMV seems logical. This would allow for rapid control of gastric motility and stomach muscle contraction in response to noxious stimuli in the blood.

Uncaging glutamate over a neuron causes a transient membrane depolarization that subsides over a period of ~500 ms. Consequently, an increased response to uncaging, as was seen here, is presumed to be the result an increase in excitability in the afferent neuron. Initial uncaging experiments indicated an increase in glutamatergic NTS neuron excitability in T1DM mice. To understand this, we first measured the intrinsic membrane properties of these neurons in both control and T1DM mice. The mouse model used here expresses EGFP in GABAergic neurons, so to increase the likelihood of obtaining responses from glutamatergic neurons, only EGFP-negative neurons were targeted for recording, though this does not imply that all neurons were glutamatergic. FGF19 produced a mixed effect on intrinsic membrane properties in these neurons, which did not appear to be dependent on hyperglycemic state. In both control and T1DM groups, FGF19 failed to alter R_in_ and RMP in approximately half of NTS neurons. In the neurons that did respond, FGF19 tended to produce relatively subtle inhibitory effects, although a small proportion of neurons were excited by FGF19. Similarly, FGF19 produced a predominately inhibitory effect on evoked action potential firing in response to positive current injections. These responses were mainly similar to our previous results in the DMV, which noted a modest inhibitory effect of FGF19 on intrinsic properties of DMV neurons in both control and T1DM groups, suggesting that the peptide may interact with a common intracellular pathway downstream from the FGFR that is not modified by hyperglycemia (6).

The effects of FGF19 on NTS intrinsic excitability run contrary to the excitation that was predicted by glutamate uncaging. Although it is possible that the small proportion of NTS neurons that were excited by FGF19 was responsible for the increase in evoked EPSCs in T1DM mice, further experiments on synaptic excitability were warranted. FGF19 significantly increased sEPSC frequency in most NTS neurons from T1DM mice, with no overall effect in control mice. This is consistent with the findings from **Figure 1** and is likely a key driver of the FGF19-induced increase in NTS to DMV excitatory transmission. Intriguingly, and in contrast to several other metabolic hormones, this effect was abolished with the addition of TTX, suggesting the involvement of intact upstream neurons (21, 62). The small decrease in mean sEPSC amplitude is likely due to an increase in frequency of lower amplitude events and not a postsynaptic mechanism, since this effect was not retained with the addition of TTX. Since FGF19 was found to increase the activity of the AP to DMV glutamatergic connection, the effect of AP input to the NTS was tested. As hypothesized, glutamate release in the NTS that was due to activity of AP neurons was increased by FGF19.

Together, these results suggest that FGF19 increases glutamatergic neurotransmission at multiple points in the DVC circuit of T1DM mice. ICV administration of FGF19 decreases blood glucose concentration in T1DM but not control mice (6, 63, 64). This suggests that the effects on blood glucose are likely to be determined by neurophysiological differences between control and T1DM groups. Although FGF19 produced intrinsic inhibition in some NTS neurons, this effect was broadly similar between both animal groups and was relatively modest. Moreover, the effects on intrinsic excitability of NTS neurons do not appear to be consequential regarding their net excitatory influence on DMV neurons in T1DM mice. Rather, the more substantial effects of FGF19 on excitatory synaptic activity in T1DM mice play a greater role in the peptide’s effect on vagal motor output. The effects on intrinsic properties of NTS neurons observed here are also consistent with our prior results showing that FGF19 produced a small decrease in sEPSC frequency in some DMV neurons from control mice (6).

In addition to neuronal input, the DVC regulates metabolism in response to humoral signals. The AP and NTS lack a fully functional blood-brain barrier due to the presence of local fenestrated capillaries, which could allow the diffusion of humoral components into the DVC that might typically be excluded (19). Additionally, NTS and DMV neurons have wide dendritic fields (61, 65) that can extend to the AP border. As such, it is likely that neurons in all three DVC nuclei can sense and respond to humoral signals. FGF19 crosses the blood-brain barrier, albeit slowly, but it is unknown whether its entry into the brain is enhanced in areas that contain fenestrated capillaries (66). Neurons in the DVC have been shown to respond to many primary metabolic hormones (21–25, 34, 67). Similar to the effects of FGF19 described here, leptin, insulin, CCK-8, and ghrelin modify glutamatergic, but not GABAergic transmission in the DVC (21, 22, 34, 62, 68). Despite this, GABAergic transmission is considered to be a primary determinant of DMV neuron activity during resting conditions, since blockade of glutamatergic transmission in this area fails to produce effects on gastric motility or pancreatic secretion in normoglycemic mice (9, 11). However, an NMDA receptor antagonist applied to the NTS attenuated jejunal nutrient sensing-mediated mechanisms of glucose production (69). Interestingly, chemogenetic silencing of GABAergic NTS neurons fails to alter blood glucose concentration in normoglycemic mice, whereas increasing GABA neuron activity in the dorsal hindbrain increases blood glucose concentration (15). This suggests that glucoregulatory DMV neurons may not show the same GABA-dominant phenotype seen with other functional DMV subgroups.

The changes in cellular excitability in the DVC seen here are consistent with the beneficial metabolic effects associated with ICV administration of FGF19. ICV FGF19 has been shown to decrease hepatic glucose production (2) and hepatic expression of G6Pase, a key enzyme required for gluconeogenesis (3). These effects can be replicated by injection of excitatory neurotransmitters into the DVC (14) or by activating DMV neurons as measured by c-fos staining (70). Additionally, a common hallmark of FGFR activation is induction of phosphorylated extracellular signal-related kinase (pERK). Insulin activation of ERK signaling in the DVC is sufficient to decrease hepatic glucose production, suggesting that FGF19 may share this mechanism (67). Injection of NMDA in the DVC lowers hepatic glucose production and this effect was prevented by a hepatic vagotomy, suggesting that the effect was mediated by the excitation of DMV neurons (14). Additionally, increasing synaptic inhibition to the DMV increases blood glucose concentration (15). Together, these results are consistent with a model in which increased excitatory input to the DMV produces a decrease in blood glucose concentration (71). Accordingly, the effects of FGF19 seen here on DVC circuit dynamics are likely to produce beneficial effects on blood glucose levels in diabetic animals.

The effects of FGF19 in the DVC found here may also modulate metabolism independently of autonomic mechanisms. Central delivery of FGF19 has been shown to decrease food intake, which could be explained by alterations in DVC neuron activity (4, 47). Similar to the effects of insulin on hepatic glucose production, satiation produced by CCK in the DVC requires induction of pERK, suggesting that FGF19 may decrease food intake *via* a shared mechanism. The NTS and AP also exhibit significant connections to other nuclei associated with regulation of ingestive behavior including the lateral parabrachial nucleus (PBN) (35, 72) and the hypothalamus (73). The experiments performed here focus on local circuitry within the DVC. As such, it is not known whether connections to other nuclei such as the PBN or hypothalamus are altered by FGF19. However, considering the significant expression of FGFR/β-Klotho throughout the NTS and AP, it is likely that FGF19 alters the activity of neurons that project centrally as well as those that participate in local vago-vagal reflexes (26, 28).

The DVC is a key metabolic regulatory area of the brain. Although the DVC is typically associated with autonomic regulation of metabolism, the AP and NTS also serve as a communication hub between the DVC and several other key nuclei. Alterations in DVC neuron activity, such as those seen here, are likely to produce profound effects on multiple aspects of metabolism. Consistent with our previous work, these results suggest that FGF19 alters parasympathetic output in T1DM mice by increasing synaptic excitability of DMV neurons. Interestingly, our findings demonstrate a role for the AP in the direct regulation of vago-vagal reflex mechanisms and that this connection can be modified by a metabolic hormone. Though not confirmed, it is possible that other metabolic hormones work similarly in this area. Overall, the electrophysiological effects seen here are consistent with the beneficial metabolic effects of ICV FGF19. Although these findings implicate a primarily autonomic mechanism, FGF19 was also found to significantly alter synaptic inputs originating from the AP and NTS. This raises the possibility that FGF19 may interact with the DVC to produce an array of beneficial metabolic effects through alteration of vagal parasympathetic output and possibly also through connections with other important metabolic regulatory nuclei.

## Data Availability Statement

The raw data supporting the conclusions of this article will be made available by the authors, without undue reservation.

## Ethics Statement

The animal study was reviewed and approved by University of Kentucky Institutional Animal Care and Use Committee.

## Author Contributions

JW designed experiments, collected data, analyzed data, prepared figures, wrote, and edited the manuscript. BS designed experiments, edited manuscript. All authors contributed to the article and approved the submitted version.

## Funding

BS is funded by the National Institutes of Health (grants R01 DK122811 and R01 DK056132).

## Conflict of Interest

The authors declare that the research was conducted in the absence of any commercial or financial relationships that could be construed as a potential conflict of interest.

## Publisher’s Note

All claims expressed in this article are solely those of the authors and do not necessarily represent those of their affiliated organizations, or those of the publisher, the editors and the reviewers. Any product that may be evaluated in this article, or claim that may be made by its manufacturer, is not guaranteed or endorsed by the publisher.
